# Randomized Trial of Piperaquine with Sulfadoxine-Pyrimethamine or Dihydroartemisinin for Malaria Intermittent Preventive Treatment in Children

**DOI:** 10.1371/journal.pone.0007164

**Published:** 2009-09-28

**Authors:** Badara Cisse, Matthew Cairns, Ernest Faye, Ousmane NDiaye, Babacar Faye, Cecile Cames, Yue Cheng, Maguette NDiaye, Aminata Collé Lô, Kirsten Simondon, Jean-Francois Trape, Oumar Faye, Jean Louis NDiaye, Oumar Gaye, Brian Greenwood, Paul Milligan

**Affiliations:** 1 Department of Parasitology and Mycology, Universite Cheikh Anta Diop, Dakar, Senegal; 2 London School of Hygiene and Tropical Medicine, London, United Kingdom; 3 Institut de Recherche pour le Developpement, Dakar, Senegal; 4 Xi'an Jiaotong University College of Medicine, Xi'an, China; 5 Ministere de la Sante, Senegal; Walter and Eliza Hall Institute of Medical Research, Australia

## Abstract

**Background:**

The long terminal half life of piperaquine makes it suitable for intermittent preventive treatment for malaria but no studies of its use for prevention have been done in Africa. We did a cluster randomized trial to determine whether piperaquine in combination with either dihydroartemisin (DHA) or sulfadoxine-pyrimethamine (SP) is as effective, and better tolerated, than SP plus amodiaquine (AQ), when used for intermittent preventive treatment in children delivered by community health workers in a rural area of Senegal.

**Methods:**

Treatments were delivered to children 3–59 months of age in their homes once per month during the transmission season by community health workers. 33 health workers, each covering about 60 children, were randomized to deliver either SP+AQ, DHA+PQ or SP+PQ. Primary endpoints were the incidence of attacks of clinical malaria, and the incidence of adverse events.

**Results:**

1893 children were enrolled. Coverage of monthly rounds and compliance with daily doses was similar in all groups; 90% of children received at least 2 monthly doses. Piperaquine combinations were better tolerated than SP+AQ with a significantly lower risk of common, mild adverse events. 103 episodes of clinical malaria were recorded during the course of the trial. 68 children had malaria with parasitaemia >3000/µL, 29/671 (4.3%) in the SP+AQ group, compared with 22/604 (3.6%) in the DHA+PQ group (risk difference 0.47%, 95%CI −2.3%,+3.3%), and 17/618 (2.8%) in the SP+PQ group (risk difference 1.2%, 95%CI −1.3%,+3.6%). Prevalences of parasitaemia and the proportion of children carrying *Pfdhfr* and *Pfdhps* mutations associated with resistance to SP were very low in all groups at the end of the transmission season.

**Conclusions:**

Seasonal IPT with SP+PQ in children is highly effective and well tolerated; the combination of two long-acting drugs is likely to impede the emergence of resistant parasites.

**Trial Registration:**

ClinicalTrials.gov NCT00529620

## Introduction

Although the incidence of malaria is declining in many parts of Africa, it remains an important public health problem, especially in poorer rural communities with limited access to health care. In Senegal, in the Niakhar demographic Surveillance area in 2007, 31% of deaths amongst children under 5 years were attributed to malaria (A Marra personal communication). In areas of seasonal malaria transmission, the burden of severe disease and mortality due to malaria is mainly among children under 5 years of age. Intermittent preventive treatment (IPT) with antimalarial drugs given to all children once a month during the transmission season can provide a high degree of protection against malaria [1]. Implementation of the intervention, organized by local health services and delivered by community health volunteers, has been highly effective in pilot studies, well accepted by communities and achieved high coverage [2]. The choice of an appropriate drug regimen is critical to the success of IPT as a public health strategy. An important consideration is the possible impact of IPT on the emergence and spread of drug resistant parasites; the drug regimen must be safe and well tolerated, and should provide sustained protection against malaria. Seasonal IPT with sulfadoxine-pyrimethamine (SP) and one dose of artesunate resulted in a 90% reduction in incidence of clinical malaria in a trial in Senegal [3]. A second trial in Senegal [4] showed that a combination of two non-artemisinin drugs with relatively long half lives (SP and amodiaquine (AQ), the latter given over three days) was more effective than SP with one or three doses of artesunate and more effective than AQ with artesunate, in preventing malaria. Very few children developed parasitaemia, so that the potential for drug resistant parasites to emerge and spread was low. Although SP+AQ was more efficacious than the artemisinin-containing regimens tested, it was associated with a higher frequency of adverse events, especially vomiting, as AQ has a bitter unpleasant taste.

Piperaquine, an antimalarial compound belonging to the 4-aminoquinolines similar in chemical structure to chloroquine and amodiaquine, has a long terminal half life which makes it especially suitable for prevention and a potential alternative to AQ for use in intermittent preventive treatment. Piperaquine was first synthesised in France by Rhone-Poulenc (13228 RP) in 1963. It was investigated for its potential in preventing malaria following oral administration at monthly or longer intervals [5]. In a large field trial in West Africa, 13228RP was given at subtherapeutic doses to children and adults at fortnightly and monthly intervals in combination with another bisquinoline compound 12494 RP, to determine the duration of protection which was found to be about 3 weeks [6] and to treat uncomplicated malaria cases [7] but its use was not pursued in Africa and Senegal continued to use chloroquine for chemoprophylaxis for children under 15 yrs until 1992 [8]. However, piperaquine was adopted in China where it had been independently synthesised in 1966. Piperaquine was used for chemoprevention in Hainan Island Province from 1968 [9]; piperaquine combined with sulfadoxine, known as Preventive No 3, was developed when some chloroquine-resistant parasites were found to be resistant to piperaquine, and this combination was used from 1969 [10], [11], [12]. Large scale trials of piperaquine for chemoprevention of falciparum and vivax malaria were conducted in several provinces. In field trials undertaken conducted in children and adults in Hainan Island province from 1972–74 [13], piperaquine alone or combined with sulfadoxine was given monthly during the main transmission season from June to September. The period of protection from malaria was reported to be about 20 days with both regimens. Side effects (headache, listlessness and dizziness) were mild, and no serious side effects were noted. Piperaquine replaced chloroquine as the recommended treatment for *falciparum* malaria in China in 1978. Over the next 14 years, 214 tons of piperaquine, equivalent to 140,000,000 adult doses, were used for mass prophylaxis and treatment.

Piperaquine has similar pharmacokinetic properties to chloroquine. Once-daily dosing of piperaquine for 3 days results in a 3- to 7-fold accumulation [14] and a long terminal half life after three daily doses of 33 days [15], making piperaquine especially suitable for chemoprevention. Dihydroartemisinin-piperaquine (DHA+PQ) has been developed as a fixed-dose combination antimalarial (Artekin) by Holleykin Pharmaceuticals, China and has been evaluated extensively as a treatment for uncomplicated clinical malaria in large clinical trials in Southeast Asia, China and Rwanda (reviewed by Myint *et al.* [16]), showing that the combination is safe, well tolerated, and highly efficacious. DHA-PQ is being used increasingly in Southeast Asia and is part of national treatment recommendations in Cambodia and Vietnam. Duocotecxin (Holley, China), containing 40 mg dihydroartemisinin and 320 mg piperaquine phosphate per tablet, is marketed in several African countries. A GMP formulation of DHA+PQ is being developed by Sigma Tau with support from the Medicines for Malaria Venture and should be licensed in 2009. An artemisinin-containing drug combination is not a natural choice for IPT because of the short half-life of artemisinins and widespread use of artemisinins could facilitate the emergence and spread of artemisinin resistant parasites damaging the usefulness of this class of drugs in treating acute cases of uncomplicated and complicated malaria. However, DHA+PQ is currently the only licensed product containing PQ available for use. We therefore compared the acceptability, efficacy and safety of combining PQ with SP and compared this combination with DHA+PQ and sulfalene-pyrimethamine plus AQ, for intermittent preventive treatment in children in a rural area of Senegal.

## Methods

The protocol for this trial and supporting CONSORT checklist are available as supporting information; see Checklist S1 and Protocol S1.

### Ethics

The study protocol was approved by the Ethics Committee of the Senegalese Ministry of Health (Conseil National de Recherche en Santé), and by the ethics committee of the London School of Hygiene and Tropical Medicine. A Data Safety Monitoring Board monitored the trial. After approval from community leaders had been obtained, the study was explained to parents or guardians of eligible children. Consent was documented by signature of the parent or guardian, or, if they could not sign, by signature of a witness.

### Study population and randomization

The study was conducted in the catchment area of Keur Soce health post in Ndoffane district, a rural area of Senegal. After meetings with health staff and community representatives, a census of 29 villages was conducted in July 2007 to enumerate all children under 5 years of age. The area was then divided into 33 circuits, each circuit including approximately 60 children to be covered by one community health worker who would deliver IPT drugs once per month. Since acceptability is difficult to assess in the formal setting of a trial, and because the method of delivery may affect compliance and acceptability, drug treatments were delivered by community workers at home, replicating as closely as possible the conditions under which IPT would be delivered routinely in Senegal. The community worker circuit was the unit of randomization to avoid contamination due to sharing of tablets within a household. Circuits were randomized to receive either SP+PQ, SP+AQ or DHA+PQ after being divided into two strata, one small stratum comprising circuits in the central village of Keur Soce in which the health post is located and where most houses have electricity, and outlying villages forming the larger second stratum. A community worker was assigned to each circuit, then the list of 33 circuits was randomized, using the random number generator in Stata version 9 (Statacorp, Texas). Randomization was checked for balance with respect to cluster size and distance from health centre and repeated until a balanced randomization was achieved. In September, each household was visited by local community health workers and trial staff to explain the study to parents and guardians of children aged 2 months to 5 years and to invite them to participate. Children who did not have a history of allergy to study drugs and whose parent or guardian provided consent were recruited and, if the child was well, the first dose of the treatment was administered. If the child was unwell, he/she was referred to the clinic, children diagnosed with malaria were treated with AQ/AS, children who did not have malaria received their IPT dose.

### Drug delivery and administration

IPT was administered on three occasions (September, October and November) covering the latter part of the malaria transmission season. Once a month, the local community worker came to the house of a study child to administer the first dose of the drug treatment. At each round, any children in the household who were not present at the initial census were included if they were aged 2–59 months and if their parents gave consent. Treatment rounds consisted of treatment over three days, the first dose was given or its administration supervised by the community worker; tablets for days 2 and 3 were left with the mother to give unsupervised. Dosing was according to age. Anthropometric data from a survey of 2000 children resident in the study area undertaken in September 2004 (K Simondon, unpublished data) were used to estimate doses by age that would avoid under-dosing and minimise over-dosing. Children in the DHA-PQ arm received Duocotexcin tablets (40 mg dihydroartemisinin and 320 mg piperaquine phosphate, Holley, China), a half tablet if aged <24 months, a whole tablet if aged 24 months or above. Children in the SP+PQ arm received piperaquine tablets (250 mg piperaquine phosphate, Shanghai Pharmaceutical Company, China), half tablet if under 12 months and a whole tablet if 12 months or over, and sulfadoxine-pyrimethamine (500 mg sulfadoxine and 25 mg pyrimethamine, Nestor Pharmaceuticals Ltd, Faridabad, India), half tablet (<12 months), a whole tablet (> = 12 months). Children in the SP+AQ arm received Dualkin (amodiaquine 200 mg half a tablet of amodiaquine (<24 months) or a whole tablet (> = 24 months), and sulfamethoxypirazyne (sulfalene) 500 mg – pyrimethamine 25 mg, Pfizer, Dakar), half a tablet (<12 months), or a whole tablet (> = 12 months). Sulfamethoxypirazyne is chemically very similar to sulfadoxine. The predicted dose ranges of these dosing schedules obtained using the anthropometric dataset were that 90% of children would receive daily doses between 9.4 and 19 mg/kg (amodiaquine); 28 to 58 mg/kg (sulfadoxine or sulfamethoxypirazyne); 1.4 to 2.9 mg/kg (pyrimethamine); 15 to 30.5 mg/kg (piperaquine phosphate in the Duocotexcin group); 14.2 to 29.2 mg/kg (piperaquine phosphate in the SP+PQ group); and 1.9 to 3.8 mg/kg (dihydroartemisinin). As a check on the actual dose received, 9 of the community workers were given scales to record child weights at each treatment round. Piperaquine was supplied by the Shanghai Pharmaceutical Company and Duocotexcin by Holley, China. Both Dualkin and Duocotexcin are licensed for treatment of uncomplicated malaria in children; the combination SP+PQ is not licensed. To ensure compliance with European regulations regarding drug quality, a sample of piperaquine tablets was tested for drug content and impurities, drug concentration was within 5% of the nominal level and analysis of impurities indicated although some impurities exceeded the ICH limit for known impurities [17], all these impurities were identified and none were of concern in terms of safety.

### Surveillance for malaria, compliance, and adverse events

Surveillance for malaria and adverse events that might have been related to drug administration was maintained from September to December 2007. Mothers or guardians of children in the study were asked to take their child to Keur Soce health post if the child became ill in any way during the period of the study. If the child had an axillary temp > = 37.5°C or a history of fever in the last 48 hours, a finger prick blood sample was taken for malaria diagnosis by rapid test and a blood film obtained to be read later. Children with a positive test were treated with AQ/AS (according to national guidelines for uncomplicated malaria), and did not receive the scheduled IPT treatment that month. Children with a negative test received the scheduled IPT treatment. In addition, one month after each treatment round, before the next dose was given, trial staff visited each child independently of the next treatment visit to check the child's health and, if the child had an axillary temperature > = 37.5°C the child was referred to the nearest dispensary and managed as described above. In September, all children were visited 4 days after the first treatment dose to record compliance with the drug regimen and to ask the mother about any adverse events, using a structured questionnaire. In October and November, a subsample of 200 children in each arm of the trial were visited to record compliance and adverse events on day 4. One month after the last treatment, all children were visited to check for malaria symptoms and to provide a finger prick blood sample for detection of malaria parasitaemia, measurement of haemoglobin concentration and investigation of the presence of parasite genotypes associated with resistance to sulfadoxine and pyrimethamine. Passive surveillance for malaria was maintained at the adjacent health post of Lamaram during the period of the trial in order to provide an estimate of malaria incidence in children who did not have IPT.

### Laboratory methods

Children with suspected malaria were diagnosed using the Paracheck-Pf® test (Orchid Biomedical Systems, Goa, India), diagnosis was confirmed if both control and test lines indicated a positive result for *P. falciparum*, the test was repeated if the control line did not appear. From the same sample, thick smear blood films were stained with Giemsa and read twice. Slides were declared negative if no parasites were seen after examining 100 microscope fields. Four drops of blood were collected on filter paper, desiccated and stored with silica gel for analysis of parasite genotype in the laboratory of parasitology of the Cheikh Anta Diop University. DNA was extracted from filter papers corresponding to the positive slides by the hot methanol method and analyzed by PCR. For the PfDHFR gene, point mutations at codons 51, 59 and 108 were detected by Mutation Specific PCR as previously described [18], [19]. For the PfDHPS gene, PCR RFLP was carried out to identify mutations at codons 437 and 540 [19], [20]. Products were analysed using 1.5% agarose gel and visualised by UV illumination after staining with ethidium bromide.

### Endpoints

The primary endpoints were the cumulative incidence of malaria (parasite density > = 300/uL with an axillary temperature > = 37.5°C or a history of fever in the previous 48 hours), and the proportion of children who experienced moderate or severe adverse events. Secondary endpoints were malaria incidence with any parasitaemia in the presence of fever or a history of fever, compliance with and acceptability of the regimen, the prevalence of parasitaemia, the mean haemoglobin concentration and the prevalence of moderate (Hb<9 g/dL) and severe anaemia (Hb<5 g/dL), and the proportion of children carrying parasite genotypes associated with resistance to sulfadoxine or pyrimethamine at the end of the transmission season (one month after the final treatment round).

### Sample size

Sample size was calculated for comparison of each treatment group to the group who received SP+AQ, the best regimen identified in the trial conducted previously in Niakhar [4]. Sample size was chosen firstly to be able to establish non-inferiority with respect to malaria incidence (we assumed an expected cumulative incidence of malaria of 5% in all groups). On the basis of previous studies, 40% of children in this population would be expected have an episode of malaria each year [3]. We chose a non-inferiority margin of 5%, and determined the sample size for 80% power and 95% confidence, and allowed for 10% loss to follow-up. For an individually randomized trial, 355 children per group would be required. Assuming an intraclass correlation (ICC) of 0.01, based on the assumption that true malaria incidence will range from 0% to 10% among clusters, and a cluster size of 60 children, then 10 clusters, i.e. 600 children, per treatment group were required. In the 2004 IPTc trial, 14% of children in the SP+AQ group experienced an adverse event in the week after IPT treatment. Similar calculations to those described above indicated 20 clusters of approximately 60 children the trial had at least 80% power to detect a 50% reduction in the incidence of adverse events.

### Statistical methods

A statistical analysis plan was written before the final database was available. For safety endpoints, all children who received at least one treatment dose were included. For efficacy endpoints both according to protocol (ATP) and intention to treat (ITT) analyses were performed. Children were included in the ATP analysis if they were given the first dose of all three treatment rounds, (regardless of whether day 2 and 3 doses were received). Children who received the first daily dose but later vomited were included in the ATP analysis, as were children who missed IPT in a particular month because they were being treated for malaria on the day IPT treatment was due. All children resident in the study area, (those enumerated in the initial census and any children who reached the age of 3 months during the trial or immigrated) and were <60 months of age at the September treatment round were included in the ITT analysis of efficacy. To take account of the cluster-randomized design, analysis was based on the (unweighted) mean (for continuous variables) or proportion (for binary variables) for each cluster [21]. Comparison of malaria incidence between the treatment groups was based on the two-sided 95% confidence interval for the risk difference [22], [23]. Confidence intervals were derived allowing for stratification [24]. Pre-specified covariates for which adjustment was made were distance from the health centre, age of the child and whether the child used a bednet (defined as usually sleeping under a treated or intact net). Adjustment for covariates was performed by the two-step method proposed by Bennett *et al.* [25] using aggregated residuals from a regression model fitted to the data for individuals. Secondary endpoints (prevalence of parasitaemia and mean haemoglobin) were analysed in a similar way, with adjustment for the same covariates. The incidence of adverse events was compared using a stratified t-test to compare cluster-level proportion of children who experienced vomiting, fever, rash, itching or headache within four days of treatment. Tests for interaction between intervention group and age group on the risk of adverse events was carried out as described by Cheung *et al.* [26], using predefined age groups (<2 years and ≥2 years). Where appropriate, age-specific risk differences were calculated; otherwise the age-adjusted risk differences are shown. The proportion of children carrying *P. falciparum* DHFR and DHPS genotypes associated with resistance to SP was estimated as the product of the probability of being slide-positive for *P. falciparum* and the probability of carrying resistance genotype if slide-positive. Standard errors were estimated from the standard errors for the two probabilities using the delta method. The chi-squared test of homogeneity was used to determine if there was an association between dose administered (mg/kg bodyweight, grouped according to quartiles) and the incidence of adverse events (mother's report of vomiting, fever, or any adverse event, recorded on day 4 after the treatment round).

## Results

### Participant flow

**Figure 1:**
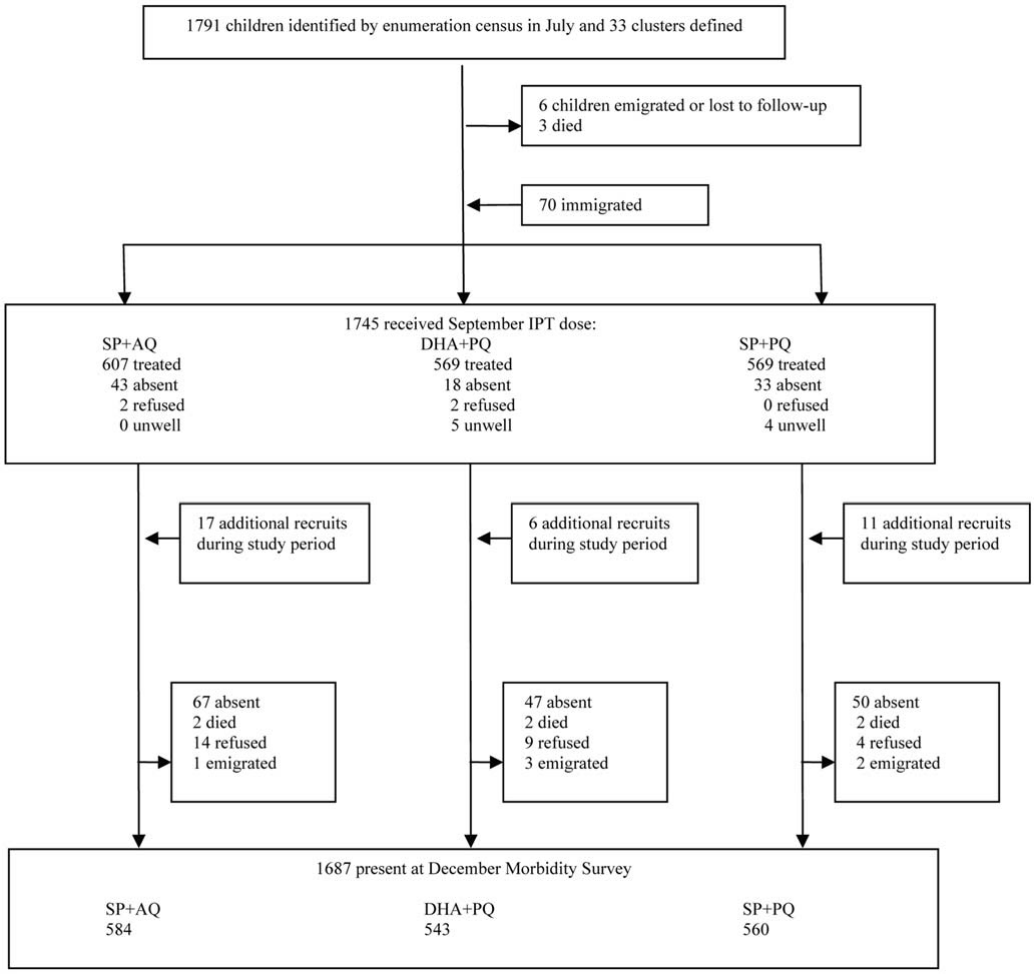
Trial Profile.

1791 children under 5 years of age were enumerated in July 2008, six were lost to follow-up, three died, and an additional 70 children were recruited by the time of the first treatment round. 1257 of these children were included in the ATP analysis. 1893 children (including 34 who were recruited after the September round) were included in the ITT analysis. Two clusters in the SP+PQ arm received SP+AQ in error in September, these clusters were excluded from the ATP analysis and included in the SP+PQ arm in the ITT analysis. 1687 children were present at the final survey in December (Figure 1). The three arms of the trial were similar in terms of the number, age and gender of children, and distance of houses from the health centre; bednet coverage (defined as the child usually sleeping under an intact or treated net) was slightly higher in the DHA+PQ arm (Table 1). The main ethnic groups in the study area were Wolof, Peulh, Toucouleur and Serere.

**Table 1:** Baseline characteristics of treatment groups.

	SP-AQ	DHA-PQ	SP-PQ
Number of children	654	598	607
Number of clusters	11	11	11
Mean cluster size (range)	59.5 (39–70)	54.5 (40–74)	55.2 (40–69)
Mean (sd) age (months)	29.3 (16.5)	30.1 (16.7)	29.3 (16.3)
Gender, % Male	48.2 (315/654)	45.3 (271/598)	47.5 (288/607)
Bednets: % sleeping under an intact or treated net	26.4 (172/651)	34.2 (204/597)	26.4 (160/607)
Mean distance (sd) of clusters from health centre	3.5 (2.5)	2.4 (2.0)	2.7 (1.9)

### Coverage of IPT doses and adherence to drug regimen

The percentage of children who received three, monthly doses of IPT from the community health-worker was similar in all groups (overall 70%, Table 2). The main reason doses were missed was the absence of families from the village at the time of the treatment round. Almost all children (98%) who received the first daily dose from a community worker were reported by the mother to have been given the second and third doses at home, and of these approximately 80% of the children were reported to have swallowed the doses without difficulty. In October and November, the proportion that swallowed all three doses without difficulty was highest in the DHA-PQ group (in this group children had to swallow only one tablet or half tablet). Among children who were weighed, the 2.5% and 97.5% percentiles of the dose per kg received (number of tablets divided by measured body weight at the time of drug administration) were pyrimethamine 1.31 to 3.20 mg/kg, sulfalene 20.1 to 52.1 mg/kg, amodiaquine 8.0 to 20.8 mg/kg/day, sulfadoxine 26.1 to 64.0 mg/kg, piperaquine (in SP+PQ group) 13.1 to 32.0 mg/kg/day, piperaquine (in DHA+PQ group) 12.6 to 34.3 mg/kg/day, dihydroartemisinin 1.57 to 4.28 mg/kg/day.

**Table 2:** Compliance with monthly IPT schedule and adherence to 3-day regimens.

	SP-AQ	DHA-PQ	SP-PQ
**Number of monthly IPT doses from health-worker**	**(N = 654)**	**(N = 598)**	**(N = 607)**
0	3.7% (24)	3.3% (20)	2.5% (15)
1	6.7% (44)	6.5% (39)	7.3% (44)
2	17% (109)	19% (112)	23% (142)
3	73% (477)	71% (427)	67% (406)
3 monthly IPT doses or malaria at time of IPT*	74% (485)	72% (433)	68% (410)
**Compliance with 3-day IPT course**			
**September**			
Given all 3 doses †	99% (685/694)	98% (546/559)	98% (443/454)
Swallowed all 3 doses †	78% (546/694)	81% (451/559)	82% (373/454)
**October**			
Given all 3 doses	95% (155/164)	98% (161/165)	94% (139/148)
Swallowed all 3 doses	70% (115/164)	88% (145/165)	76% (113/148)
**November**			
Given all 3 doses	94% (138/147)	95% (133/140)	93% (125/134)
Swallowed all 3 doses	71% (105/147)	93% (130/140)	78% (104/134)

* includes children who were seen at the monthly visit but did not receive IPT because they had malaria and were treated with an antimalarial.

† Denominator is children given IPT and followed up four days later. For September, treatment groups are of different sizes because two clusters received SP-AQ instead of SP-PQ.

### Malaria incidence

**Figure 2:**
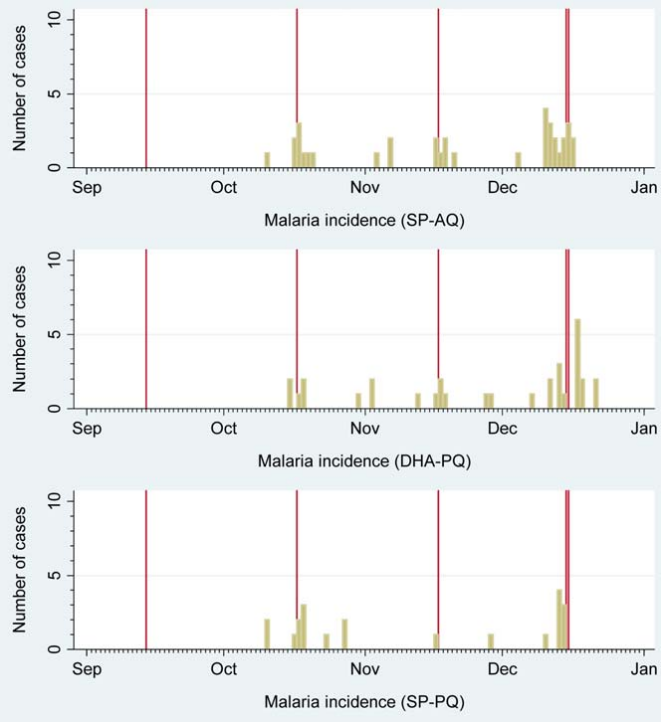
Malaria incidence during the study period. Incidence of first episode malaria associated with parasitaemia at any parasite density in the study area between September-December 2007. Single red lines indicate median date of the IPT rounds in September, October and November. Double red line indicates the median date of the December morbidity survey. One child had a second malaria episode in mid-November and 13 children had a second malaria episode detected at the survey in December (not shown).

103 episodes of malaria were recorded in 89 children (cumulative incidence 4.7%, 89/1893, Table 3) 14 of whom had a second episode. 34 of these cases were detected through passive surveillance maintained at the health centre, the remaining cases were detected through monthly active surveillance visits. Malaria was suspected as the cause of death for 2 children who died at the start of November, one in the SP-AQ group and one in the DHA-PQ group. The October IPT dose had been refused in both cases. 48 children had malaria detected during the 3 month period when IPT was given and an additional 41 new cases were detected at the morbidity survey in December. No child had more than two episodes of malaria. Incidence of malaria episodes over the study period is shown in figure 2. 68 children had malaria with parasite density >3000/uL. The risk of malaria was similar in all three groups (the point estimates of malaria risk were slightly lower in the group that received SP-PQ). 95% and 99% confidence intervals for the risk difference between SP-PQ and SP-AQ excluded the pre-specified inferiority margin of 5% in both the ATP and ITT analyses. The 95% confidence interval for the risk difference between DHA-PQ and SP-AQ also always excluded 5%. Both piperaquine containing regimens are thus non-inferior to SP-AQ at the 95% confidence level.

**Table 3:** Cumulative incidence of malaria during the study period.

	SP-AQ	DHA-PQ	SP-PQ
Malaria (>3000/µl), Intention to treat	4.3% (29/671)	3.6% (22/604)	2.8% (17/618)
Adjusted risk difference, %* (95%CI)	Reference	−0.47% (−3.25%, 2.31%)	−1.18% (−3.64%, 1.28%)
(99%CI)		(−4.28%, 3.34%)	(−4.55%, 2.19%)
Malaria (>3000/µl), According to protocol	4.1% (20/485)	3.5% (15/433)	2.4% (8/339)
Adjusted risk difference, %* (95%CI)	Reference	−0.64% (−3.18%, 1.9%)	−1.53% (−4.22%, 1.15%)
(99%CI)		(−3.98%, 2.69%)	(−5.06%, 1.99%)
Malaria (any parasitaemia) Intention to treat	5.4% (36/671)	5.3% (32/604)	3.4% (21/618)
Malaria (any parasitaemia) According to protocol	5.0% (24/485)	4.9% (21/433)	2.7% (9/339)

* Adjusted for age, bednet use and distance from health centre.

ICC for malaria >3000, 0.007, and for malaria any parasitaemia, 0.018.

In Lamaram health post, adjacent to the study area, there were an estimated 117 cases of malaria in children under 5 years of age during the period of the trial, detected by passive surveillance, from a catchment population estimated to include 1088 children, an incidence rate of 11%.

### Adverse events

Three children died before the September treatment round; six children died during the intervention period. Deaths occurred at home and the most likely cause of death was determined by verbal autopsy. A pertussis outbreak occurred in the study area during the trial; two girls in the SP-PQ group, aged 5 and 6 months, who died in October, were suspected to have had pertussis. Two girls, one aged 3 months in the SP-AQ group and one aged 4 months in the DHA-PQ group died in September; death was attributed to febrile diarrhoea. Two boys (one in the SP-AQ group aged 48 months and one in the DHA-PQ group aged 27 months) died from a febrile illness suggestive of malaria at the start of November.

No drug-related severe adverse events were reported. When the mother or carer was asked about adverse events four days after the first treatment round, fever, vomitting and headache were more commonly reported in the SP+AQ group than in either of the PQ groups (Table 4). Rash and itching were less commonly reported and incidence was similar in all groups. For two symptoms, vomiting and fever, there was a significant interaction between treatment and age, reflecting the fact that in the SP+AQ group, incidence of both symptoms was more common among children over 2 yrs of age than among children under 2 years old, while in the DHA-PQ and SP-PQ groups, these symptoms were similar in each age group (fever) or slightly more common among under-2 yr olds (vomiting). Results were similar after the second and third treatment rounds (data not shown).

**Table 4:** Incidence of solicited adverse events reported by mother or carer four days day after the first administration of IPT.

	SP-AQ (N = 694)	DHA-PQ (N = 559)	SP-PQ (N = 454)
**Vomitted**	218 (31%)	76 (14%)	76 (17%)
<24 months	26% (74/282)	17% (36/210)	21% (42/196)
Risk difference (95%CI)	Reference	−8.4 (−18, 1.6)	−5.6 (−16, 5)
≥24 months	35% (144/412)	12% (40/348)	13% (34/258)
Risk difference (95%CI)	Reference	−22.7 (−32, −14)	−22.1 (−32, −13)
**Fever**	219 (32%)	98 (18%)	89 (20%)
<24 months	28% (79/282)	20% (42/210)	19% (37/196)
Risk difference (95%CI)	Reference	−5.9 (−16.5, 4.7)	−8.3 (−19.6, 3)
≥24 months	34% (140/412)	16% (56/348)	20% (52/258)
Risk difference (95%CI)	Reference	−17 (−28, −5.5)	−13 (−25, −1.1)
**Rash**	14 (2%)	3 (0.5%)	5 (1.1%)
Risk difference (95%CI)	Reference	−1.2 (−3.1, 0.6)	−0.8 (−2.8, 1.1)
**Itching**	27 (3.9%)	7 (1.3%)	8 (1.8%)
Risk difference (95%CI)	Reference	−2.7 (−5.5, 0.1)	−2.4 (−5.4, 0.6)
**Headache**	87 (13%)	32 (5.7%)	18 (4%)
Risk difference (95%CI)	Reference	−6.7 (−13.2, −0.1)	−8.7 (−15.6, −1.7)

Intraclass correlation coefficients for each outcome were as follows: vomiting 0.067, fever 0.078, rash 0.024, itching 0.039, headache 0.072. Risk differences were adjusted for age, or presented stratified by age group when there was a signification interaction with age.

### Association of adverse events with dose

When incidence of adverse events was examined in relation to the dose received in the subset of children who were weighed, there was a significant association with the incidence of vomiting for amodiaquine, but there was no evidence of an association with the risk of other adverse events, and no association of the risk of adverse events with dose for other drugs. Of 312 children who received SP+AQ and for whom weights were recorded, 33% received a daily dose of AQ >15 mg/kg (the prescribed number of tablets per day divided by the weight in kg recorded the day treatment was given), of these 41% (43/104) were reported to have vomited during three days of treatment, compared to 26% (55/208) who received doses <15 mg/kg, an odds ratio of 2.0 (95%CI 1.2–3.2, P = 0.008).

### Prevalence of malaria parasitaemia and markers of SP resistance

4.4% of protocol compliant children (56/1260) were parasite positive at the December survey by microscopy (table 5). Gametocytes were detected in only 11 children (4 in the SP+AQ group, 5 in the DHA+PQ group and 2 in the SP+PQ group), an overall prevalence of 0.9% (11/1260).

**Table 5:** Prevalence of parasitaemia and of molecular markers of resistance to SP at the end of the transmission season.

	SP-AQ	DHA-PQ	SP-PQ
Percentage slide positive	5.3% (22/413)	4.8% (19/395)	3.3% (15/452)
Percentage of children (95%CI)* carrying:			
dhfr triple mutation (51, 59, 108)	3.6% (0.7, 19.3)	1.5% (0.6, 3.7)	1.8% (0.5, 7.3)
dhps-437	1.8% (0.3, 9.7)	1.9% (0.8, 4.4)	1.5% (0.3, 6.4)
quadruple mutation	1.2% (0.2, 6.9)	0.7% (0.3, 2.2)	0.4% (0.02, 6.0)
Percentage of parasite-positive individuals carrying:			
dhfr triple mutation(51, 59, 108)	67% (12/18)	31% (4/13)	56% (5/9)
dhps-437	33% (6/18)	39% (5/13)	44% (4/9)
quadruple mutation	22% (4/18)	15% (2/13)	11% (1/9)

* estimated as the product of the probability of being positive for *P.falciparum* and the probability of carrying resistant genotype if positive for *P.falciparum*, with standard errors estimated from the standard errors for the two probabilities. The intra-class correlation coefficient for being slide positive was 0.088.

The overall number of children carrying *P. falciparum* parasites with the triple *Pfdhfr* mutations associated with resistance to pyrimethamine was very low in all groups (Table 5). Point estimates were slightly lower in the groups that received piperaquine. 21 of the 40 samples that were typed (53%) were positive for the dhfr triple mutation and 15 (38%) for the dhps-437 mutation associated with resistance to sulphonamides.

### Anaemia

27% of children (332/1222) enrolled in the study were moderately anaemic (Hb<9 g/dL) in December; 14 children were severely anaemic (Hb<5 g/dL) (table 6). Prevalence of anaemia and of severe anaemia was greater among children who received DHA-PQ than among those who received SP+AQ. Mean haemoglobin concentration was lower among children who received DHA+PQ than in the SP+AQ group (a difference in Hb concentration, adjusted for covariates, of 0.46 g/dL, 95% CI 0.1 to 0.81 g/dL, in the ATP analysis and 0.38 (0.03 to 0.73) in the ITT analysis).

**Table 6:** Prevalence of anaemia and mean haemoglobin concentration at the end of the malaria transmission season.

	SP-AQ	DHA-PQ	SP-PQ
Anaemia (Hb<9 g/dl)	23.3% (125/537)	34.3% (170/495)	27% (136/503)
Severe anaemia (Hb<5 g/dl)	0.7% (4/537)	2% (10/495)	1% (5/503)
Mean haemoglobin concentration (g/dL)*	10.07	9.63	9.85
Difference in mean Hb (g/dL) (95%CI)	Reference	0.38 (0.03, 0.73)	0.06 (−0.33,0.45)

* The ICC for Hb concentration was 0.041.

Hb concentration in the SP+PQ group was similar to that in the SP+AQ group.

## Discussion

IPT in pregnant women, infants or children requires an effective, well-tolerated drug regimen as many subjects who receive treatment will not need it. IPT currently relies on SP, but as resistance to SP is high in some countries where IPT is indicated alternative drugs are required. Long-acting regimens are needed to minimise dosing frequency and maximise cost effectiveness. The long terminal half life and excellent tolerability of piperaquine make it a suitable choice for IPT. This is the first study of its use for malaria prevention in Africa since it was initially investigated for this purpose in West Africa in the mid 1960's. PQ in combination with SP or DHA was well tolerated, and was as effective as SP+AQ in preventing malaria. Because of the previous demonstration of the efficacy of IPT in children in Senegal it was not considered appropriate to include a placebo group in this study and it is, therefore, not possible to determine the efficacy of the PQ containing combinations. However, comparison of the results obtained in this study with those of the control group included in the 2002 study of IPTc in the same area (cumulative incidence of malaria 41%, [3] compared with incidences of 3.6% and 2.8% in the PQ containing regimens in the current study) suggests that efficacy was high. The incidence of malaria in the area adjacent to the trial site, detected by passive surveillance, was 11%, this maybe compared to the incidence of 34 cases (2%) that were detected by passive surveillance in the trial population, again compatible with high efficacy of the regimens used in the trial.

A previous trial in Senegal showed that SP+AQ was more effective than artemisinin combinations in preventing malaria in children but that the use of AQ was associated with a higher incidence of mild and moderate adverse events, of which fever, vomiting, and headache were the most commonly reported [4]. In the present study, these symptoms were significantly less common in the DHA+PQ and SP+PQ groups than in the SP+AQ group.

The prevalence of asexual parasitaemia and of gametocytaemia was very low in all treatment groups at the end of the transmission season, reducing the potential impact of IPT on the spread of drug resistant parasites. Less than 2% of children treated with SP+PQ or DHA+PQ carried the triple *Pfdhfr* mutation associated with resistance to pyrimethamine. These results are compatible with the longer terminal half life of PQ (over 1 month) compared to AQ (about 1 week), which may be important in preventing recrudescence of SP-resistant parasites in children treated with SP, and in preventing establishment of infections with parasites with SP-resistant genotypes. SP remains effective against malaria in most parts of West Africa; limiting its use to IPT in combination with PQ could extend the useful life of both drugs. A further advantage of SP+PQ over SP+AQ is that its longer action may provide some protection for children who miss monthly doses.

The prevalence of PfDHFR triple mutation was lower in this study than in children who received IPT with SP+AQ or SP+AS in the two previous trials in the same area [3], [4]. Since 2004, artemisinin combinations (AS+AQ) have been used for treatment of uncomplicated malaria in Senegal. The possible impact of this policy on the prevalence of parasite genotypes associated with resistance to SP should be monitored.

DHA+PQ was similar to SP+PQ in terms of efficacy and tolerability but DHA is not an ideal drug for IPT, it's rapid elimination from the body means that any preventive action of the combination will rely solely on PQ increasing selection pressure for PQ resistance. DHA may ensure rapid clearance of any parasitaemia present at the time of treatment, but we found no evidence that DHA+PQ was more effective than SP+PQ in terms of clinical outcomes. It is probably not advisable to use the same drug combination for treatment of children with acute clinical malaria and for prevention. In communities where artemether+lumefantine or AQ+artesunate are first line treatments for malaria, SP+PQ may be a good combination for prevention, reducing drug pressure on the artemisinins whose major advantage is their rapid mode of action. In communities where DHA+PQ is used for treatment, SP+AQ may be an acceptable combination for prevention although associated with a higher incidence of minor side effects than SP+PQ. Where SP resistance is more marked, the use of SP in IPT regimens may be less effective, there is therefore a need for alternative long-acting antimalarials to be developed.

Anaemia was more common in the group that received DHA+PQ, this may be a chance finding, previous studies have not reported anaemia associated with DHA, although artemsinins can case transient reduction in reticulocyte count [27].

Dosing by age resulted in some children receiving doses in excess of the recommended dose for their body weight. In the SP+AQ group children who were overdosed were more likely to report adverse events; it is likely that this was due to AQ rather than SP (overdosing with SP+PQ was not associated with an increased risk of adverse events). It may, therefore, be possible to improve the tolerability of SP+AQ by improving the accuracy of dosing. Compliance with the second and third daily doses, given unsupervised by the mother, was slightly better with the single-tablet regimen (DHA+PQ) than with the other regimens. Development of fixed dose coformulated tablets for IPT may improve compliance. Single dose regimens would also be preferable but the long action of PQ may require accumulation of concentration over three days.

Many antimalarial treatments, including SP+AQ and SP+CQ, have been adopted for clinical use and included in national treatment policies without the formal programme of pharmaceutical development now required by regulatory authorities for new drugs or drug combinations, dosing schedules having been established through clinical practice. Pharmacokinetic data for piperaquine became available only comparatively recently [14], [15]. PQ has a similar chemical structure to CQ and AQ, both of which can be safely and effectively used in combination with SP, so an interaction of PQ with SP is therefore highly unlikely. Preclinical toxicological studies of PQ plus sulfadoxine were conducted in China [28], [29], and a number of clinical trials of this combination have been reported [30], [13]. Nevertheless pharmacokinetic studies of SP+PQ in children may be required before widespread use of the combination could be recommended.

This trial was not blinded, as use of dummy tablets would not have been compatible with the pragmatic design. However, malaria diagnosis relied on use of objective diagnostic tests and laboratory staff who read blood films did not have access to treatment assignments. Trial staff and parents were aware of the child's treatment group which could have influenced reporting and assessment of adverse events, nevertheless as the trial was conducted in an operational setting, the data obtained may give a fair reflection of the acceptability of the regimens if they were to be used routinely.

Coverage of treated bednets was low in the study area. Most trials of IPTc have been conducted in areas where ITN coverage is low and consequently there is limited information about the interaction of IPT and ITNs, but it is likely that IPT would add to the protection provided by bednets. In a randomized trial of ITNs and chemoprophylaxis with dapsone/pyrimethamine, using a nested factorial design, malaria incidence was significantly reduced in the children who received chemoprophylaxis and ITNs compared to children using ITNs alone [31]. A trial designed to estimate the additional benefit of IPT in children using ITNs is currently being conducted in Burkina Faso and Mali (clinicaltrials.gov NCT00738946).

Two children who missed their October dose of IPT died in early November, probably from malaria. Where IPT is introduced efforts should be made to encourage families to ensure that their children receive each monthly dose. Home management for malaria, whereby antimalarial treatment is available (presumptively or after a positive RDT) at home or in the vicinity of the home for children with a febrile illness, may be able to prevent such deaths. Home management for malaria has been evaluated in several countries [32] but studies of its use in combination with IPT are needed.

The results of this trial support an increasing body of evidence that seasonal IPT in children is a safe and highly effective strategy for malaria prevention. SP+PQ is cheap and well tolerated and its use could optimise the effectiveness of seasonal IPT. High coverage and excellent compliance can be achieved when IPT is delivered through local health services. Large scale implementation of IPT is now required to demonstrate effectiveness of IPT as public health strategy.

## Supporting Information

Checklist S1 CONSORT checklist
(0.06 MB DOC)

Protocol S1 Trial Protocol
(0.76 MB PDF)
