# Supplementary material for: Randomized Trial of Piperaquine with Sulfadoxine-Pyrimethamine or Dihydroartemisinin for Malaria Intermittent Preventive Treatment in Children
Source: PLoS One. 2009 Sep 28;4(9):e7164. doi: 10.1371/journal.pone.0007164 (PMC2747010; doi:10.1371/journal.pone.0007164)
Supplement: Protocol S1 — Trial Protocol (0.76 MB PDF) [file pone.0007164.s002.pdf]

Randomized trial of the safety, acceptability and efficacy of three drug regimens for seasonal intermittent preventive treatment for malaria.

Principal Investigator
Dr Badara Cisse, Department of Parasitology, Universite Cheikh Anta Diop, Dakar, Senegal

Sponsor: London School of Hygiene and Tropical Medicine

Funding: EDCTP

Summary
The purpose of this trial is to compare the acceptability, efficacy and safety of alternative drug regimens for use for seasonal Intermittent Preventive Treatment to prevent malaria in children. After meetings with health staff and community representatives, 1800 children aged 2 months to 5 years, who do not have a history of allergy to study drugs and whose parents give consent, will be recruited by house to house visits, and randomized to receive IPT with one of three regimens on three occasions during the transmission season, (in September, October and November): sulfadoxine-pyrimethamine (SP) plus amodiaquine, show to be highly effective for IPT in a recent trial; SP plus piperaquine, used for malaria prophylaxis in China for many years; or Artekin (a combination of piperaquine with an artemisinin). Each treatment is a 3-dose regimen over 3 days, the first dose will be supervised and the other 2 doses given by the carer. One month after each treatment round, children will be visited at home to check for malaria symptoms, children with fever or a history of fever in the last 48 hours will be asked to give a finger prick blood sample for malaria diagnosis. One month after the last treatment all children will be asked to give a finger prick blood sample for parasitology and haemoglobin, axillary temperature will be measured, and the child's carer will be interviewed about compliance and adverse events. The endpoints will be the cumulative incidence of malaria, the proportion of children experiencing moderate and severe adverse events, compliance with and acceptability of the regimen, the prevalence of parasitaemia, and the proportion of children carrying parasite genotypes associated with resistance to sulfadoxine or pyrimethamine at the end of the transmission season. Since acceptability is difficult to assess in the formal setting of a trial, and because the method of delivery may affect compliance and acceptability, drug treatments will be delivered by community workers replicating the conditions under IPT would be delivered routinely in Senegal. Treatments will be administered at home by community workers, each worker covering a circuit of approximately 60-80 children. The community worker circuit will be the unit of randomization, for simplicity in the field to minimise allocation errors, and to avoid contamination due to sharing of tablets within a household. The study will be conducted in the catchment area of Ker Soce poste de sante in Ndoffane district in Senegal.

Background

In areas of seasonal malaria transmission the burden of severe disease and mortality due to malaria is mainly among children under 5 years of age. Intermittent preventive treatment (IPT) with antimalarial drugs given to all children once a month during the transmission season is a promising new strategy for malaria prevention. Seasonal IPT with sulfadoxine-pyrimethamine (SP) and one dose of artesunate resulted in a 90% reduction in incidence of clinical malaria in a recent trial in Senegal (Cisse et al., Lancet 2006). An important consideration is the possible impact of seasonal IPT on the emergence and spread of drug resistant parasite genotypes, the choice of drug regimen is therefore critical. A second trial in Senegal (Sokhna et al., submitted) showed that a combination of two non-artemisinin drugs with relatively long half lives (SP and amodiaquine (AQ) over three days) was more effective than SP with one or three doses of artesunate and more effective than AQ with artesunate, in preventing malaria, and very few children developed parasitaemia, so that the potential for drug resistant genotypes to emerge and spread was low. Although SP+AQ was more efficacious than the artemisinin-containing regimens tested, it was associated with a higher frequency of adverse events, especially vomiting, and AQ has a bitter unpleasant taste, and therefore we have concerns about the acceptability of AQ for widespread use for IPT. Relatively minor adverse events may be

acceptable when a life-threatening illness is being treated, but may not be acceptable when drugs are given on a large scale for prevention. The perception by the community that a drug is associated with adverse events could derail implementation of IPT as a control strategy. Therefore it is important to select a drug regimen that is not only effective but safe and acceptable to the community.

Methods

The study will be conducted in the catchment area of Ker Soce poste de sante in Ndoffane district in a rural area of Senegal. After meetings with health staff and community representatives, parents or guardians of children aged 2 months to 5 years will be invited to participate, and children who do not have a history of allergy to study drugs, with consent from a parent or guardian, will be recruited in house to house visits, and then in September allocated (according to the randomization by community worker) to receive IPT with one of the three regimens on three occasions during the transmission season.

Study drugs and dosing

Duocotexcin: 0.5 tablets if the child is <2yrs old, 1 tablet if >=2yrs
Piperaquine: 0.5 tablets <1yr, 1 tablet if >=1yr
SP: 0.5 tablets if <1yr, 1 tablet if >=1 yr
Amodiaquine 200mg tab (dualkin): 0.5 tablet if child <2yrs, 1 tablet if >=2yrs

Children in the DHA-PQ arm will receive Duocotexcin tablets (40mg dihydroartemisinin and 320mg piperaquine phosphate, Holley, China), a half tablet if aged <24 months, a whole tablet if aged 24 months or above. Children in the SP+PQ arm will receive piperaquine tablets (250mg piperaquine phosphate, Shanghai Pharmaceutical Company, China), half tablet if under 12 months and a whole tablet if 12 months or over, and sulfadoxine-pyrimethamine (500mg sulfadoxine and 25mg pyrimethamine), half tablet (<12months), a whole tablet (>=12 months). Children in the SP+AQ arm will receive Dualkin (amodiaquine 200mg half a tablet of amodiaquine (<24 months) or a whole tablet (>=24 months), and sulfamethoxypirazyne (sulfalene) 500mg – pyrimethamine 25mg, Pfizer, Dakar), half a tablet (<12 months), or a whole tablet (>=12 months).). Sulfamethoxypirazyne is chemically very similar to sulfadoxine. The predicted dose ranges of these dosing schedules obtained using the anthropometric dataset were that 90% of children would receive daily doses between 9.4 and 19 mg/kg (amodiaquine); 28 to 58 mg/kg (sulfadoxine or sulfamethoxypirazyne); 1.4 to 2.9 mg/kg (pyrimethamine); 15 to 30.5 mg/kg (piperaquine phosphate in the Duocotexcin group); 14.2 to 29.2 mg/kg (piperaquine phosphate in the SP+PQ group); and 1.9 to 3.8 mg/kg (dihydroartemisinin). As a check on the actual dose received, 9 of the community workers will be given scales to record child weights at each treatment round. Piperaquine to be supplied by the Shanghai Pharmaceutical Company and Duocotexcin by Holley, China. To ensure compliance with European regulations regarding drug quality, a sample of tablets will be tested for drug content and impurities by Sigma Tau, Rome.

Sample size
Sample size has been calculated for comparison of each treatment group to the group who receive SP+AQ3, the best regimen identified in the trial conducted previously in Niakhar (Sokhna et al 2004), firstly to establish non-inferiority with respect to malaria incidence (we have assumed an expected cumulative incidence of malaria of 5% in all groups). Typically 50%-70% of children in this population will have an episode of malaria each year, a drug which reduces this to 10%, which is safe and acceptable, if preferable to a drug which can reduce incidence to 5% but has problems of adverse events and poor compliance. We therefore chose a non-inferiority margin of 5%, and determined the sample size for 80% power and 95% confidence, and allowed for 10% loss to follow-up. In an individually randomized trial 355 per group are required. Assuming intraclass correlation (ICC) of 0.01, based on the assumption that true malaria incidence will range from 0% to 10% among clusters, and a cluster size of 60 children, then 10 clusters, i.e. 600 children, per treatment group are required. For the incidence of adverse events, in the 2004 trial 14% of children in the SP+AQ3 group experienced an adverse event in the week after IPT treatment. Similar calculations indicate this sample size has at least 80% power to detect a 50% reduction in the incidence of adverse events.

Study duration and location

June to December 2007. Keur Soce, Ndoffane district of Senegal.

Procedures

There will be an interview to explain the study and seek consent, conducted by trial staff and community workers. Once a month (Sept, Oct, Nov) a local community worker will come to the house to administer the first dose of the drug treatment. One month after each treatment, before the next dose is given, trial staff will visit, independently of the next treatment visit, to check the childs health, and if the child has axillary temp >=37.5°C or a history of fever in the last 48 hours a finger prick blood sample will be taken for malaria diagnosis by rapid test and blood film to be read later. Children with a positive test will be treated with AQ/AS (according to national guidelines for uncomplicated malaria). Children with signs of severe malaria, or any other illness, will be referred to the health post. All children will be visited 4 days after the first treatment round to record compliance and adverse events, using a structured questionnaire to interview the carer. To monitor compliance and adverse events 4 days after the October and November treatment rounds, a subsample of 200 children in each arm will be visited. One month after the last treatment, all children will be visited and asked to provide a finger prick blood sample for malaria parasitaemia, haemoglobin, and parasite genotyping.

Ethics

The drugs and drugs combinations to be used in this pilot study are all registered for the treatment of malaria in young children but SP+PQ and AS+PQ have not been previously used for IPT in Africa. Thus, children enrolled in the study will all be seen 4 days after the first treatment dose to check for adverse events. No invasive investigations will be performed; blood samples will be collected by finger prick. The trial will be conducted according to the standard GCP with care being taken to protect staff as well as study recipients. Finger prick is the only procedure that might cause distress. Our medical staff are, however, well trained to perform this operation causing the minimum of discomfort.

Community consent will be sought from community leaders. For taking finger prick blood samples, documented oral consent from a parent or guardian in the presence of at least one witness will be sought. The message given to the parents will focus on the fact that although children will receive chemoprophylaxis against malaria they are strongly advised to bring their children for examination at the nearest health post if the child is unwell at any time, where they will receive free treatment.

Medical supervision is available through the government nurses who live at the health post Keur Soce, in addition the project will employ one full time clinican and one nurse who will be based at the health post. Severe adverse events will be reported to the PI within 24 hours. All children will be visited 4 days after the start of the first treatment round to check for adverse events. Kaolack hospital,18km away, is a large well-equipped regional hospital.

The proposal will be submitted to the Ethics Committee of the Senegalese Ministry of Health (Conseil National de Recherche en Santé) and the London School of Hygiene and Tropical Medicine.

A DSMB will be appointed to monitor the trial. Insurance liability is covered through LSHTMs insurance.

Data processing and confidentiality

Names and other personal identifiers on the study forms will not be entered into computer files. Paper forms will be stored in locked filing cabinets in the trial office. Computerised data will be identified using a unique numeric ID number.

Dissemination of findings:

A Working Group for studies of IPT in children has been established in Senegal chaired by Prof O Gaye, this group includes representatives from all key partners including Ministry of Health, the group will meet in the first quarter of 2008 to share results of the study. At the same time, with staff of the health post and the district medical officer, we will visit the

communities in Ndoffane to share the key findings. The results will be prepared for publication in a peer reviewed journal.

Experience of Principal Investigators

Dr Badara Cisse holds an EDCTP fellowship, he is an experienced clinical scientist with 16 years of clinical research experience at the Pasteur Institute and IRD, Senegal, and attached to the Gates Malaria Partnership (2002 to 2006). He will be responsible for coordination and day to day running of the trial, training of field staff, and supervision of health post staff. Paul Milligan is Reader in Epidemiology and Medical Statistics in EPH, will be responsible for design and coordination of the trial.

Date de la visite I__I__I I__I__I I__I__I Village ___________________________________I___I___I Hameau ___________________________________I___I___I

Etude pilote (phase IV) randomisé en simple aveugle comparant l'efficience de trois combinaisons d'antipaludiques potentielles candidats pour le traitement préventif intermittent saisonnier délivré par des relais communautaires

N° CC	**Nom du CC**	**Nom de l'enfant**	Identification	**Sexe**	**Date de naissance**	**Nom de la mère**	**Nom du père**	**Raison de non recensement**
I__I__I			I__I__I I__I__I I__I__I__I	I___I	I__I__I I__I__I I__I__I<br>ou age approx I__I. I__I			
I__I__I			I__I__I I__I__I I__I__I__I	I___I	I__I__I I__I__I I__I__I<br>ou age approx I__I. I__I			
I__I__I			I__I__I I__I__I I__I__I__I	I___I	I__I__I I__I__I I__I__I<br>ou age approx I__I. I__I			
I__I__I			I__I__I I__I__I I__I__I__I	I___I	I__I__I I__I__I I__I__I<br>ou age approx I__I. I__I			
I__I__I			I__I__I I__I__I I__I__I__I	I___I	I__I__I I__I__I I__I__I<br>ou age approx I__I. I__I			
I__I__I			I__I__I I__I__I I__I__I__I	I___I	I__I__I I__I__I I__I__I<br>ou age approx I__I. I__I			
I__I__I			I__I__I I__I__I I__I__I__I	I___I	I__I__I I__I__I I__I__I<br>ou age approx I__I. I__I			
I__I__I			I__I__I I__I__I I__I__I__I	I___I	I__I__I I__I__I I__I__I<br>ou age approx I__I. I__I			
I__I__I			I__I__I I__I__I I__I__I__I	I___I	I__I__I I__I__I I__I__I<br>ou age approx I__I. I__I			
I__I__I			I__I__I I__I__I I__I__I__I	I___I	I__I__I I__I__I I__I__I<br>ou age approx I__I. I__I			

Poste de santé: ____ Enquêteur: ____ Page I___I___I

PHARMACOVIGILANCE (SUIVI a JOUR 4)

Village ______________________________ Nom du CC______________________________

Nom de l'enfant ______________________________ Date de naissance : |__|__| |__|__| |__|__|__|__| Age |__|__| mois Sexe |__| M/F

Nom de la mère ______________________________ Date de visite: |__|__| |__|__|__| |__|__|__|__|

Poids de la mère : |___|___|.|___| Poids mère + enfant : |___|___|.|___| Tour de bras 1 : |___|___|.|___| Tour de bras 2 : | __|___|.|___|

L'enfant est il présentement malade ou a-t-il été malade ces 3 derniers jours? Oui |__| Non |__| Nsp |__|

Si Oui, cochez chaque symptôme et précisez sa durée et sa sévérité:

**Symptômes**	**Cochez**	**Durée: Jours**	**Heures**	**Sévérité* (1, 2 ou 3)**
Fièvre (rapportée par la mère)	|__|	|__|	|__|__|	|__|
Maux de tête	|__|	|__|	|__|__|	|__|
Somnolence	|__|	|__|	|__|__|	|__|
Vertiges	|__|	|__|	|__|__|	|__|
Douleurs abdominales	|__|	|__|	|__|__|	|__|
Perte d'appétit	|__|	|__|	|__|__|	|__|
Nausées/vomissements	|__|	|__|	|__|__|	|__|
Diarrhée	|__|	|__|	|__|__|	|__|
Toux	|__|	|__|	|__|__|	|__|
Eruptions cutanées	|__|	|__|	|__|__|	|__|
Prurit (grattage)	|__|	|__|	|__|__|	|__|
Ictère (yeux jaunes)	|__|	|__|	|__|__|	|__|
Autres: spécifiez________________________	|__|		|__|__|	|__|

L'enfant a t il vomi ce jour ou lors de ces 3 derniers jours ? Oui |__| Non |__| Nsp |__|

Si oui, combien de fois |__|__|

Commentaires: décrivez n'importe quel symptôme, qu'il soit modéré ou sévère, présenté par l'enfant et précisez en détails l'action prise pour juguler le mal. ____

____

Température axillaire: |___|___|. |___|°C L'enfant se sent-il/elle bien aujourd'hui? |__| *Oui /Non/NSP*

Symptômes lors des dernières 48 heures:

	Oui/Non/Nsp		*Oui/Non/Nsp*
Fièvre	|__|	Vomissements	|__|
Toux	|__|	Diarrhée	|__|
Difficulté respiratoire	|__|	Eruption	|__|
Convulsions	|__|	Autres Symptômes	

Si l'enfant est fébrile, (température ≥38.0°C) ou antécédents de fièvre dans les 48 dernières heures, faire si possible un prélèvement.

La goutte épaisse a-t-elle été faite? *Oui/Non* |__| Si oui, indiquez le résultat (nombre de *Plasmodium falciparum* par µl): __________

Si non, pourquoi? ____

Un TDR a-t-il été fait ? *Oui/Non* |__| Si oui, indiquez le résultat : Positif/Négatif/Ininterprétable |__|

Une goutte épaisse sur papier buvard a-t-elle été recueillie ? *Oui/Non* |__|

* Sévérité: 1= mineure (le symptôme n'empêche pas le jeu habituel), 2= modérée (le symptôme empêche le jeu comme habituellement) et 3= sévère (l'état de l'enfant nécessite ou a nécessite une consultation médicale)

PHARMACOVIGILANCE (SUIVI a JOUR 4)

Village ________________________________ |__|__| Nom du CC________________________________ |___|___|___|

ID |__|__| |__|__|__| |__|__|__| Nom de l'enfant ________________________________ Age |__|__| mois Sexe |__| M/

Nom de la mère ____

Date de visite: |__|__| |__|__| |__|__|__|__| L'enfant est il présent ? Oui |__| Non |__|

Si Non, précisez pourquoi il est absent ____

Expliquez que nous voulons savoir ce que pensent les parents, particulièrement les mères, de l'administration des médicaments aux enfants. Vous devez interroger la personne qui traitait l'enfant dans la concession.

L'enfant est il présentement malade ou a-t-il été malade ces 3 derniers jours? Oui |__| Non |__| Nsp |__|

Si Oui, cochez chaque symptôme et précisez sa durée et sa sévérité:

Symptômes	Cochez	Durée: Jours	Heures	Sévérité* (1, 2 ou 3)
Fièvre (rapportée par la mère)	|__|	|__|	|__|__|	|__|
Maux de tête	|__|	|__|	|__|__|	|__|
Somnolence	|__|	|__|	|__|__|	|__|
Vertiges	|__|	|__|	|__|__|	|__|
Douleurs abdominales	|__|	|__|	|__|__|	|__|
Perte d'appétit	|__|	|__|	|__|__|	|__|
Nausées/vomissements	|__|	|__|	|__|__|	|__|
Diarrhée	|__|	|__|	|__|__|	|__|
Toux	|__|	|__|	|__|__|	|__|
Eruptions cutanées	|__|	|__|	|__|__|	|__|
Prurit (grattage)	|__|	|__|	|__|__|	|__|
Ictère (yeux jaunes)	|__|	|__|	|__|__|	|__|
Autres: spécifiez__________________________		|__|	|__|__|	|__|

L'enfant a t il vomi ce jour ou lors de ces 3 derniers jours ? Oui |__| Non |__| Nsp |__|

Si oui, combien de fois |__|__|

Commentaires: décrivez n'importe quel symptôme, qu'il soit modéré ou sévère, présenté par l'enfant et précisez en détails l'action prise pour juguler le mal.

____

Quelle action a été prise ?____

Si l'enfant est malade, référer le au poste de santé de keur soce ou informer le médecin de terrain de projet.

Avez-vous donné du TPI à votre enfant ce mois? Oui |__| Non |__| NSP |__|

Si oui, précisez la date mentionnée sur la carte _____/_____/_________

Si non, pour quelle raison l'administration n'a pas été faite ?________________________________

* Sévérité: 1= mineure (le symptôme n'empêche pas le jeu habituel), 2= modérée (le symptôme empêche le jeu comme habituellement) et 3= sévère (l'état de l'enfant nécessite ou a nécessite une consultation médicale)

Nous voudrions maintenant avoir vos analyses sur le TPI administré à votre enfant :

	1ere dose<br>J1 (a domicile par l'asc)		
L'enfant a-t-il reçu le TPI administré par l'agent de santé communautaire ?	|__| Oui/Non/Nsp		
Si oui, l'enfant a-t-il: ***(cochez la réponse)***<br>Bien avalé le médicament ?<br>Avalé le médicament mais l'a aussitôt rendu?<br>Refusé de prendre le médicament?	 |__| |__| |__|		
L'enfant a-t-il vomi après prise du médicament ?	|__| Oui/Non/Nsp		
Si oui, la dose a-t-elle été re-administrée ?	|__| Oui/Non/Nsp	2eme dose<br>J2 (a domicile par la mère)	3eme dose<br>J3 (a domicile par la mère)
Avez-vous administré les 2nd et 3eme doses ?		|__| Oui/Non/Nsp	|__| Oui/Non/Nsp
Si non, pour quelle raison?<br>*(Mentionnez la raison dans la case)*			
Si oui, l'enfant a-t-il: ***(cochez la réponse)***<br>Bien avalé le médicament ?<br>Avalé le médicament mais l'a aussitôt rendu?<br>Refusé de prendre le médicament?		 |__| |__| |__|	 |__| |__| |__|

Demander à voir les sachets utilisés

Reste t il des médicaments dans un sachet? |__| Oui |__| Non |__| sachet(s) jeté(s) ou perdu(s)

Si oui, estimez le nombre de comprimés restants (par exemple 1 et ½): ____________________________

Pourquoi n'avez-vous pas utilisé ces médicaments? ____

Qu'allez vous faire avec les comprimés restants? ____

A propos des comprimés, pensez vous que l'enfant :

a trouvé que les comprimés de TPI étaient d'un mauvais goût? |__| Oui/Non/Nsp

L'enfant a-t-il craché le traitement? |__| Oui/Non/Nsp

Projet EDCTP
Questionnaire d'évaluation finale
Keur Socé 2007

1. Identification *Inclut **TOUS** les enfants ayant reçu au moins une dose de TPI*

Date de la visite: _______/_______/___________/

Nom du Village **et** du Hameau: ____

Nom du chef de concession____

Nom du chef de ménage____

Nom de la mère ____

Nom de l'enfant ____ ID : |___|___|___|___|___|___|___|___|___|

Date Naissance : ____/____/________ ou Âge |____|____| années |____|____| mois Sexe:|____| M /F

L'enfant vit-il dans le village depuis sa naissance : |____| Oui/Non/Nsp, Si non, depuis combien d'années vit l'enfant dans le village : |____|____| mois

2. Anthropométrie & Hémoglobinémie, Parasitémie & Papier filtre

Poids mère: |___|___|.|___| Poids mère+enfant: |___|___|.|___| TB 1 : |___|___|.|___| TB 2 : |___|___|.|___| T : |___|___|.|___|

TDR : |____| Oui/Non G.E : |____| Oui/Non Taux Hb (g/dL) : |____|____|.|____| Papier filtre : |____| Oui/Non

3. Moustiquaires *Demander le lit où l'enfant dort d'habitude et inspecter la moustiquaire*

L'enfant a-t-il dormi sous moustiquaire la nuit dernière ? |___| Oui /Non/Nsp

L'enfant dort-il d'habitude sous une moustiquaire ? |___| Oui /Non/Nsp *(par exemple, pendant l'hivernage)*

Si oui, cette moustiquaire a t-elle été traitée au moins 1 fois lors les 12 derniers mois? |___| Oui/ Non/ Nsp

Peut-elle être enfouie sous le matelas? |__| Oui /Non

Est-elle intacte (<5 trous) |__| Oui/Non

4. Doses TPI administrées *Adressez-vous à la mère ou à la tutrice de l'enfant. Inspectez la carte TPI de l'enfant*

Carte TPI vue ? |__| Oui/Non

Si oui mentionnez le numéro d'identification de la carte TPI ID : |___|___|___|___|___|___|___|

Si non une carte TPI lui a t elle été délivrée cette année ? |__| Oui/Non

Mentionnez les traitements TPI reçus chaque mois et si cela n'a pas été le cas, demandez la raison

Mois	Traitement		
Septembre	|__| Oui/Non/Nsp	Date :	si non, précisez la raison |__| 1, 2, 3, 4, 5, 6, 7, 8
Octobre	|__| Oui/Non/Nsp	Date :	si non, précisez la raison |__| 1, 2, 3, 4, 5, 6, 7, 8
Novembre	|__| Oui/Non/Nsp	Date :	si non, précisez la raison |__| 1, 2, 3, 4, 5, 6, 7, 8

1=enfant absent mais dans le village, 2=enfant en voyage, 3=mère ou tutrice absente, 4=enfant trop malade 5=réaction adverse la de la précédente administration 6=enfant trop jeune, 7=refus des parents,, 8=autre, spécifiez ____

5. A propos de la mère ou de la tutrice *Adressez-vous à la personne qui administrait le TPI à l'enfant*

Nom de celle qui s'occupe de l'enfant ________________________ - __________________ Sexe |___| M/F Âge |___|___| années

Quelle est la relation avec l'enfant ? |___| *1=Mère, 2=Père, 3=Sœur, 4=Grand-mère, 5=Tante, 6=Autre:* ___________

Quel est le statut matrimonial de cette personne? |___| *1=Marié 2=Célibataire 3=Veuve 4=Divorcée*

Cette personne a t elle été à l'école coranique? |___| Oui/Non si oui, combien d'années d'école au total? |____|____| années

Cette personne a t elle été à l'école française? |___| Oui/Non si oui, combien d'années d'école au total? |___|___| années

Cette personne a t elle été alphabétisée? |___| Oui/Non si oui, combien d'années d'école au total? |___|___| années

A t il été nécessaire de recueillir l'aval d'une tierce personne avant administration du TPI à l'enfant ? |___| Oui/Non

Si oui, qui est cette personne? |___| *1=Mère 2=Père 3=Sœur 4=Grand-mère 5=Tante 6=Autre: spécifiez*

Quelles sont les principales sources de revenues de la mère ou de la tutrice ? |___|

1=Femme au foyer 2=agriculture 3=pêche 4=commerce en détails 5=affaires 6=infirmière 7=éducatrice 8=employée de maison 9=étudiante 10=sans occupation 11=fonctionnaire 12=autre Spécifiez _________________

Reçoit t elle une assistance financière d'une personne qui vit à l'extérieur de la concession ? |__| Oui/Non

Si oui, combien en CFA le mois dernier ____ (Novembre 2007)

6. A propos du ménage

Quelqu'un dans le ménage possède t il du bétail ? |__| Oui/Non :

Si oui, Combien de : bovins ____ ovins ____ caprins ____ chevaux ____ ânes ____

Combien de ces items existent dans le ménage ? *(Inscrivez le nombre approprié dans chaque case, ignorez lorsque l'item n'existe pas)*

Radio	I___I	Mobylette/moto	I___I	Charrette	I___I
TV	I___I	Lit métallique ou en bois	I___I	Mobile	I___I
Magnétoscope	I___I	Vélo	I___I	Voiture	I___I
Montre	I___I				

La mère ou la tutrice est elle sous la responsabilité du chef de ménage? |__| Oui/Non

- Si Non, la mère ou la tutrice possède t elle du bétail ? |__| Oui/Non
- Si oui, Combien de : bovins ____ ovins ____ caprins ____ chevaux ____ ânes ____

Combien de ces biens personnels existent dans le ménage ? *(Inscrivez le nombre approprié dans chaque case, ignorez lorsque l'item n'existe pas)*

Radio	I___I	Mobylette/moto	I___I	Charrette	I___I
TV	I___I	Lit métallique ou en bois	I___I	Mobile	I___I
Magnétoscope	I___I	Vélo	I___I	Voiture	I___I
Montre	I___I				

Maison familiale ou une maison en location	I___I	*1= louée, 2 = familiale*
Type de construction	I___I	*1=terre battue (TB), 2= Ciment, 3= TB + ciment, 4= Ciment + peinture*
Type de plafond	I___I	*1= étain, 2=chaume*
Type de plancher	I___I	*1= ciment, 2=TB*

Le ménage possède t il (si oui, cochez): |__|=eau courante |__|=électricité |__|=téléphone fixe |__| =chasse d'eau |__|=fosse |__|=énergie solaire.

Qu'allumez-vous pour la cuisine ? (si oui, cochez): |__|=bois de brousse |__|=bois acheté |__|=butane |__|=électricité |__|=bouse de vache |__|=charbon autre ________________________________

7. À propos de l'enfant

Avez-vous vu l'enfant aujourd'hui ? |___| oui/non Si non, pourquoi n'avez-vous pas vu l'enfant : ______________________
____

L'enfant est t il en bonne santé aujourd'hui ? |___| oui/non Si non, quel est le problème : ____________________________
____

Sil vous plait, remplir les cases ci-dessous et évaluer l'intensité des signes ou symptômes selon la classification ci après :

0=normal,
1=événement bien toléré, ne causant pas d'inconfort et n'empêchant pas les activités quotidiennes,
2=événement qui perturbe l'enfant et l'empêche de jouer ou de se déplacer comme d'habitude,
3=événement qui retient l'enfant alité.

	Intensité	Durée en jours (1 si début aujourd'hui)
Fièvre	|___|	|___|___|
Nausées	|___|	|___|___|
Vomissement	|___|	|___|___|
Douleur abdominale	|___|	|___|___|
Perte d'appétit	|___|	|___|___|
Diarrhée	|___|	|___|___|
Eruption cutanée	|___|	|___|___|
urticaire	|___|	|___|___|
Jaunisse	|___|	|___|___|
Malaise	|___|	|___|___|
Difficulté respiratoire	|___|	|___|___|
Toux	|___|	|___|___|
Agité	|___|	|___|___|
	Intensité	Durée en jours (1 si début aujourd'hui))
Autre	|___|	|___|___|
Autre	|___|	|___|___|
Autre	|___|	|___|___|

Examen physique

	Normal (O/N)	(Si non, pourquoi)
ORL	|___|	___
Pleuro pulmonaire	|___|	___
Cardio-vasculaire	|___|	___
System nerveux central	|___|	___
Foie / Rate	|___|	___
Gastro-entérologie	|___|	___
Peau	|___|	___
Squelette / articulations	|___|	___
Autres signes:	|___|	___

Existent-ils des antécédents d'allergies aux sulfamides (SP)? |___| Oui/Non

Histoire des vaccinations: Copiez les dates de vaccination sur sa réplique en page 4. S'il n'existe pas de carte de vaccination ou si la carte est difficile à lire ou s'il s'agit d'un duplicata, posez alors les questions suivantes *:*

Demandez à voir la carte de vaccination de l'enfant

L'enfant a t-il été vaccine à la naissance ou lors de son premier contact avec le poste de santé ? |___| Oui/Non

L'enfant a t-il été vaccine à l'âge de 3 mois ? |___| Oui/Non, si oui, combine de fois ? |___|

L'enfant a t-il reçu une vaccination orale ? |___| Oui/Non si oui, combine de fois ? |___|

L'enfant a t-il reçu une ou vaccination à l'âge de 9 mois ? |___| Oui/Non

Note à l'attention des relais:

Vaccination à la naissance ou lors du 1er contact avec le poste de santé *(BCG, Polio1 et Hépatite B1)*

Cicatrice sur le haut du bras gauche *(BCG)*

Vaccinations à 3 mois *(DTP, HIB)*

Vaccination orale *(Polio)*

Vaccinations à 9 mois *(Rougeole et Fièvre Jaune)*

Nom enquêteur ou enquêtrice : ___

Consentement informé

Mme / Mr,
Votre enfant nommé ____ est inclus dans le projet sur le traitement préventif intermittent que mène le service de parasitologie médicale de l'université cheikh anta diop de Dakar. A ce fait, nous avons déjà recueilli votre consentement au mois de septembre dernier.
Le projet a été dans l'ensemble un franc succès avec, comme attendu, une importante réduction des cas de paludisme. Mais pour une évaluation complète de ce projet, il nous faut faire un prélèvement à tous les enfants inclus (quelques gouttes de sang prélevées sur le bout d'un doigt). Ce prélèvement quoi que négligeable nous permettra cependant d'analyser plusieurs paramètres. Nous serons ainsi a mesure de savoir si votre enfant a assez de sang ou non. Lorsqu'une anomalie est dépistée, le traitement idoine sera immédiatement et gratuitement administré.
Dans l'attente que vous donniez encore votre consentement pour ce prélèvement, nous vous renouvelons notre gratitude. N'hésitez pas a me contacter ou a vous adressez au superviseur de terrain, Mr Ernest Faye, pour toute interrogation ou complément d'information.

Dr Badara Cissé
Principal investigateur
Service de Parasitologie Médicale
Faculté de Médecine, UCAD
BP 5005 Dakar Fann
Téléphone : fixe 338 25 19 98 mobile 776 38 64 51

Nom de l'interlocuteur ou interlocutrice : ____

Nom du témoin : ____

Date de visite : _____/_____/__________/

Nom Village : ___________________ Nom hameau : ___________________ Registre Communautaire N^0: |___|___|

Etude pilote (phase IV) randomisée en simple aveugle comparant l'efficience de trois combinaisons d'antipaludiques potentielles candidats pour le traitement préventif intermittent saisonnier délivré par des relais communautaires

ID	Nom de l'enfant	Sexe	CC	Age (mois)	Habitué(e) MI O/N/NSP	MI Nuit dernière O/N/NSP	Etat MI	Traitement MI	Médicament 1	Médicament 2
|___| |___|___| |___|___|___|										
|___| |___|___| |___|___|___|										
|___| |___|___| |___|___|___|										
|___| |___|___| |___|___|___|										
|___| |___|___| |___|___|___|										
|___| |___|___| |___|___|___|										
|___| |___|___| |___|___|___|										
|___| |___|___| |___|___|___|										
|___| |___|___| |___|___|___|										
|___| |___|___| |___|___|___|										
|___| |___|___| |___|___|___|										

Cluster I__I__I ID relais I__I__I Initiales I__I__I__I Numéro de la balance I__I__I Date de visite I__I__I I__I__I I__I__I

Projet EDCTP Keur Socé /Diagnostic et suivi de la morbidité

1. Identification

Date : ____/____/________

Nom de l'enfant:__________________________________ ID |___| |___|___| |___|___|___| Sexe: |___| *M /F*

Date Naissance : ____/____/________ Age : |___|___| années |___|___| mois

Nom de la Mère : ____

Nom du Village et du Hameau : ____

2. Examen clinique et para clinique

Température axillaire: |___|___|. |___|^{o}C L'enfant se sent-il/elle bien aujourd'hui? |__| *Oui /Non/NSP*

Symptômes lors des dernières 48 heures:

	Oui/Non/Nsp		*Oui/Non/Nsp*
Fièvre	|__|	Vomissements	|__|
Toux	|__|	Diarrhée	|__|
Difficulté respiratoire	|__|	Eruption	|__|
Convulsions	|__|	Autres Symptômes	

Si l'enfant est fébrile, (température $\geq 37.5^{0}C$) ou antécédents de fièvre dans les 48 dernières heures, faire si possible un prélèvement. La goutte épaisse a-t-elle été faite? *Oui/Non* |__|
Si oui, indiquez le résultat (nb *Pf* par µl): __________ Si non, pourquoi? ________________________________

Un TDR a-t-il été fait ? *Oui/Non* |__| Si oui, indiquez le résultat : Positif/Négatif/Ininterprétable |__|
Si non, pourquoi? ____

Une goutte épaisse sur papier buvard a-t-elle été recueillie ? *Oui/Non* |__|

3. Diagnostic

|__| Paludisme
|__| Infection des voies aériennes sup.
|__| Otite moyenne
|__| Infections cutanée
|__| Infection pleuro pulmonaires
|__| Infection urinaire
|__| Méningite
|__| Septicémie
|__| Gastroentérite
|__| Malnutrition
Autres, précisez ____

4. Traitement délivré

1 ________________________ 3 ________________________

2 ________________________ 4 ________________________

L'enfant a-t-il/elle été référé(e) au centre de santé ? *Oui/Non* |__|

5. Nom et fonction du personnel soignant : ____

Préciser aux parents que l'enfant doit être ramené au poste de santé si son état ne s'améliore pas.
